# Causal mechanisms proposed for the alcohol harm paradox—a systematic review

**DOI:** 10.1111/add.15567

**Published:** 2021-05-25

**Authors:** Jennifer Boyd, Olivia Sexton, Colin Angus, Petra Meier, Robin C. Purshouse, John Holmes

**Affiliations:** 1 School of Health and Related Research, University of Sheffield, Sheffield, UK; 2 MRC/CSO Social and Public Health Sciences Unit, University of Glasgow, Glasgow, UK; 3 Department of Automatic Control and Systems Engineering, University of Sheffield, Sheffield, UK

**Keywords:** Alcohol consumption, alcohol-related harm, causal mechanisms, disadvantage, health inequalities, morbidity, mortality, socio-economic position

## Abstract

**Background and Aims:**

The alcohol harm paradox (AHP) posits that disadvantaged groups suffer from higher rates of alcohol-related harm compared with advantaged groups, despite reporting similar or lower levels of consumption on average. The causes of this relationship remain unclear. This study aimed to identify explanations proposed for the AHP. Secondary aims were to review the existing evidence for those explanations and investigate whether authors linked explanations to one another.

**Methods:**

This was a systematic review. We searched MEDLINE (1946–January 2021), EMBASE (1974–January 2021) and PsycINFO (1967–January 2021), supplemented with manual searching of grey literature. Included papers either explored the causes of the AHP or investigated the relationship between alcohol consumption, alcohol-related harm and socio-economic position. Papers were set in Organization for Economic Cooperation and Development high-income countries. Explanations extracted for analysis could be evidenced in the empirical results or suggested by researchers in their narrative. Inductive thematic analysis was applied to group explanations.

**Results:**

Seventy-nine papers met the inclusion criteria and initial coding revealed that these papers contained 41 distinct explanations for the AHP. Following inductive thematic analysis, these explanations were grouped into 16 themes within six broad domains: individual, life-style, contextual, disadvantage, upstream and artefactual. Explanations related to risk behaviours, which fitted within the life-style domain, were the most frequently proposed (*n* = 51) and analysed (*n* = 21).

**Conclusions:**

While there are many potential explanations for the alcohol harm paradox, most research focuses on risk behaviours while other explanations lack empirical testing.

## INTRODUCTION

Alcohol accounts for 5.3% of deaths and 5.1% of the burden of disease and injury globally [1]. However, alcohol-related harms (e.g. deaths, illnesses and hospitalizations due partly or wholly to alcohol) are not equally distributed across socio-economic positions (SEP)—the social and economic factors that determine an individual’s position in society [2].

Disadvantaged groups suffer from higher rates of alcohol-related hospital admissions and deaths compared with advantaged groups, despite reporting similar or lower average levels of consumption [3,4]. For example, in the United Kingdom, the proportion of people in the highest SEP group drinking more than 4/3 (45%) or 8/6 (23%) units per day is almost double compared to the lowest SEP (22 and 10%, respectively) [5]. Despite this, the alcohol-specific mortality rate among the most deprived is 5.5 times higher [6]. This relationship, termed the alcohol harm paradox (AHP), is found internationally, including in the United Kingdom [4], Australia [7], the Netherlands [8] and Finland [9] and across measures of SEP (e.g. social grade, income, education, car ownership, employment and housing tenure) [10]. Prior to 1980, findings suggest a clear dose–response relationship between alcohol consumption and alcohol-related hospitalization and mortality, irrespective of SEP [11–13]. However, in the last 40 years the AHP has become a consistent and long-standing finding [14]. Despite this, there is a paucity of research attempting to understand the underlying causes of the AHP.

Several reviews and meta-analyses have described socio-economic differences in alcohol-related harms based on existing evidence or available survey data [3,15–19]. However, only a subset also focused upon the contribution of alcohol consumption to this relationship, measured as average consumption (e.g. grams or units weekly, monthly or yearly) or drinking patterns (how often and how much people drink) [3,17,18]. This evidence highlights that neither average alcohol consumption nor heavy drinking patterns can explain differences in alcohol-attributable outcomes between SEP groups. At best, heavy drinking occasions partially attenuate the link between SEP and hospitalizations or mortality by 15–30% [3]. Put simply, the most disadvantaged consistently suffer disproportionate risks of harm from their alcohol consumption when compared to their advantaged counterparts, which is not only a health burden on society but contributes to increasingly widening health inequalities [20].

Empirical studies of the AHP have largely focused upon proximal individual-level factors as potential explanations. The role of unrecorded alcohol consumption has, to an extent, been investigated, and results suggest that under-reporting is similar across socio-economic groups [21]. Cross-sectional studies have also tested differences in drinking patterns, behavioural clustering and drinking histories [21,22]. Although there is evidence that low SEP groups tend to have heavier drinking patterns [21,22] and engage in multiple risky health-related behaviours [21], fewer studies go on to test the degree to which life-style risk factors explain differences in alcohol-related harm. One study highlighted that the rate of alcohol-attributable mortality and hospital admissions was three times higher for the most disadvantaged compared with the most advantaged; this association remained after adjusting for weekly consumption and heavy drinking occasions, and it was only slightly attenuated after further adjusting for body mass index (BMI) and smoking [4]. While investigation of life-style factors is prominent, other potentially fruitful avenues of explanation, such as social and economic causes (e.g. social support, housing and employment), have been neglected.

Substantial socio-economic gradients in health exist across countries and contexts [23,24]. There is a critical need for evidence to support public health policies that tackle not only behaviour, but also the broader social determinants of health to mitigate the AHP. This study aimed to review explanations for the paradox put forward in relevant scientific literature. Secondary aims were to review the existing evidence for or against these explanations, and to explore how authors combine different explanations to shed light upon potential relationships between different causal factors. To our knowledge, this is the first review to collate explanations for the AHP.

## METHODS

### Search strategy

We followed the Preferred Reporting Items for Systematic Reviews and Meta-Analyses guidelines (PRISMA) [25]. The protocol for this study can be found at: http://doi.org/10.13140/RG.2.2.25606.60489. MEDLINE (1946 – January 2021), EMBASE (1974–January 2021) and PsycINFO (1967–January 2021) were searched to identify peer-reviewed literature on the topic of the AHP or studies that investigated the relationship between alcohol-related harm, socio-economic position and alcohol consumption. An extensive list of search terms was used (see Supporting information, Table S1) to capture the themes of alcohol (e.g. alcohol adj3 drink*) and socioeconomic factors (e.g. disadvantage*). Given the large number of results returned during test searches, further specifications were made by focusing upon papers with alcohol in the title, and some exclusory terms were included (e.g. NOT therapeutics). Terms were tailored dependent upon database requirements. For grey literature, Google and Google Scholar were searched, and this was supplemented via expert identification of relevant reports (C.A.).

### Inclusion and exclusion criteria

The population, exposures, comparisons, outcome and study designs (PECOS) criteria for inclusion are listed in Table 1. Studies were included if they: (i) were full papers published in English and (ii) explicitly explored the AHP OR investigated the relationship between: alcohol-related harm, socio-economic position and alcohol consumption (Table 1). We focused upon high-income Office for Economic Cooperation and Development (OECD) countries as classified by the World Bank [26], primarily due to differences in alcohol environments between high- and low–middle-income countries, e.g. greater availability of informally produced alcohol in low–middle-income countries [27]. A range of study designs were eligible for inclusion. Systematic reviews and meta-analyses were included, as it is equally possible to extract ‘explanations’ for the paradox from these studies. However, intervention and treatment studies were outside the scope of this review. Additionally, empirical studies which analysed data exclusively collected pre-1980s were excluded.

### Screening

All records were imported to EndNote Online and duplicates were removed. Titles and abstracts were screened to identify papers matching the inclusion criteria. Full-text versions of the papers were then screened to determine inclusion. Initial screening was carried out by one reviewer (J.B.). A second reviewer (O.S.) then randomly screened a sample of the included studies (*n* = 20) to validate that papers were correctly included. There was no disagreement between reviewers regarding inclusion.

### Data extraction

Data from the papers were extracted by one reviewer (J.B.). A second reviewer (O.S.) independently assessed the accuracy of data extraction for a sample of the included studies (*n* = 20). In the case of disagreement both reviewers referred to the paper in question, and a consensus was reached. A data extraction matrix was developed, which included characteristics of the studies (design, year of data collection and location), participants (age, target population and sample size), measures (unit of analyses, SEP, alcohol consumption and alcohol harm measures) and outcomes (main findings and explanations for the AHP). Both tested and hypothetical explanations were extracted. ‘Explanations’ were any reasons identified from the empirical results or proposed by the authors which explain why alcohol-related harm outcomes were worse for those of a low SEP. Explanations were commonly taken from the results and discussion sections of empirical papers or the main body of other types of included paper. Hypothetical explanations were extracted verbatim. The evidence for these explanations was also extracted from included primary research or from authors citing other research findings when proposing an explanation.

### Quality assessment

Quality appraisal of the included studies was conducted by one researcher (J.B.) to assess risk of bias. The AXIS critical appraisal tool [28], CASP qualitative, CASP systematic review, CASP cohort study and CASP case–control study checklists [29] were used depending upon the study design. Commentaries, author replies, discussion papers and reports were not critically appraised. Overall, the quality of included papers was assessed as good. More information on critical appraisal can be found in the Supporting information, Table S2.

### Analysis

Descriptive summary statistics were used to describe search results and study characteristics. An inductive thematic approach was taken to analyse the explanations provided by included papers. This aimed to group explanations within broader themes. Explanations were coded and initially analysed by one researcher (J.B.) in consultation with co-authors (R.P. and J.H.). In the instance where an author meaningfully linked multiple explanations in the text, this was recorded as a connection. A narrative synthesis of the findings providing evidence for or against the extracted explanations was also conducted.

## RESULTS

### Descriptive analysis

**Figure 1:**
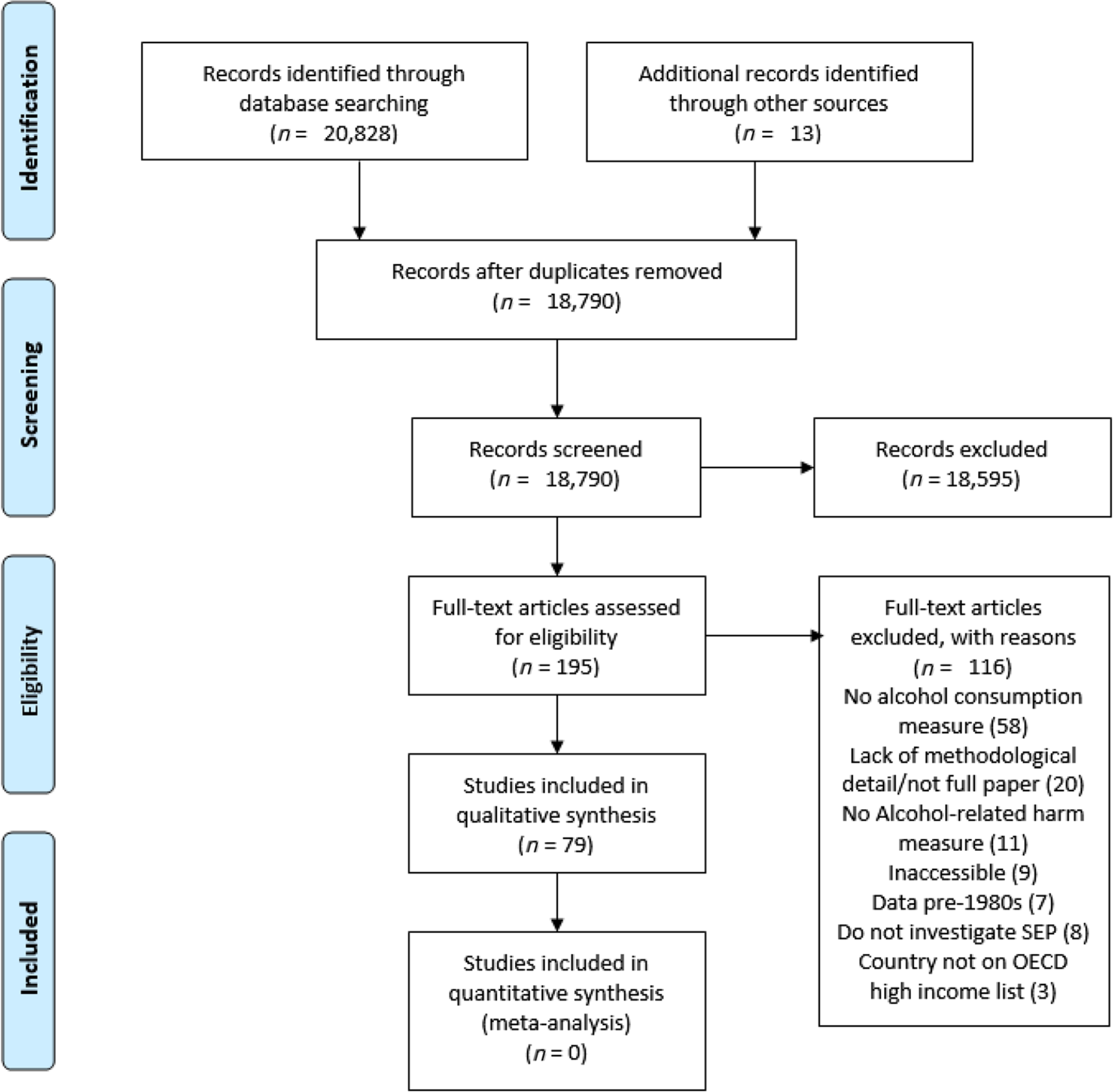
Preferred Reporting Items for Systematic Reviews and Meta-Analyses (PRISMA) flow diagram

A search of electronic databases returned 20 828 records. A further 13 records were identified from the grey literature. Total records reduced to 18 790 following de-duplication. Of the 18 790 records, following title and abstract screening, 195 were selected for full-text screening and 79 of these met the inclusion criteria for data synthesis (Fig. 1). Attempts to retrieve inaccessible papers were made through the search databases, University Library services and Google Scholar. Study characteristics are displayed in Table 2.

The largest number of papers came from the United Kingdom (*n* = 27). Other countries providing several papers included the United states, Sweden, Australia, New Zealand, Finland, France, Denmark, Canada, the Netherlands and Norway. Some studies were set at a continental (e.g. Europe) or global level. Of the included empirical studies, cohort (*n* = 26), cross-sectional (*n* = 21), case–control (*n* = 4) and qualitative (*n* = 2) designs were employed. One used both cross-sectional and longitudinal data. The included reviews and meta-analyses (*n* = 5) contained a total of 238 studies. Commentaries (*n* = 6), debate/discussion papers (*n* = 4) and reports (*n* = 10) were also included.

Empirical studies covered the general population (*n* = 37), patients only (*n* = 7), young adults (*n* = 6), men only (*n* = 2), adults with long-term health conditions (*n* = 1) and military conscripts (*n* = 1). The existence of the alcohol harm paradox was explicitly explored in 39 of the empirical studies. Of the identified papers, only seven included explicit theoretical discussion.

Of the empirical studies, the majority used at least one quantity/frequency measure of alcohol use (*n* = 36). Other measures included hazardous consumption, heavy drinking episodes, per-capita consumption, alcohol biomarkers and blood alcohol concentration (Table 2). Measures of SEP included individual-level (e.g. education) and area-level deprivation measures (Table 2). Most studies used physical health harm outcomes, including deaths, hospitalizations or disease states wholly and/or partially attributable to alcohol (*n* = 36). Other harm outcomes included negative alcohol-related consequences and alcohol use disorder or dependence (Table 2).

### Evidence of the AHP

Only three of the included empirical studies found that those of a lower SEP had higher alcohol consumption which then led to increased harm, two of which were specifically focused upon pancreatitis [30–32]. Therefore, the evidence base generally supported the existence of the AHP (*n* = 36, including three meta-analyses of a total of 72 studies); excess harm among those of lower SEP could not be explained by the volume of alcohol consumed.

### Thematic analysis

**Figure 2:**
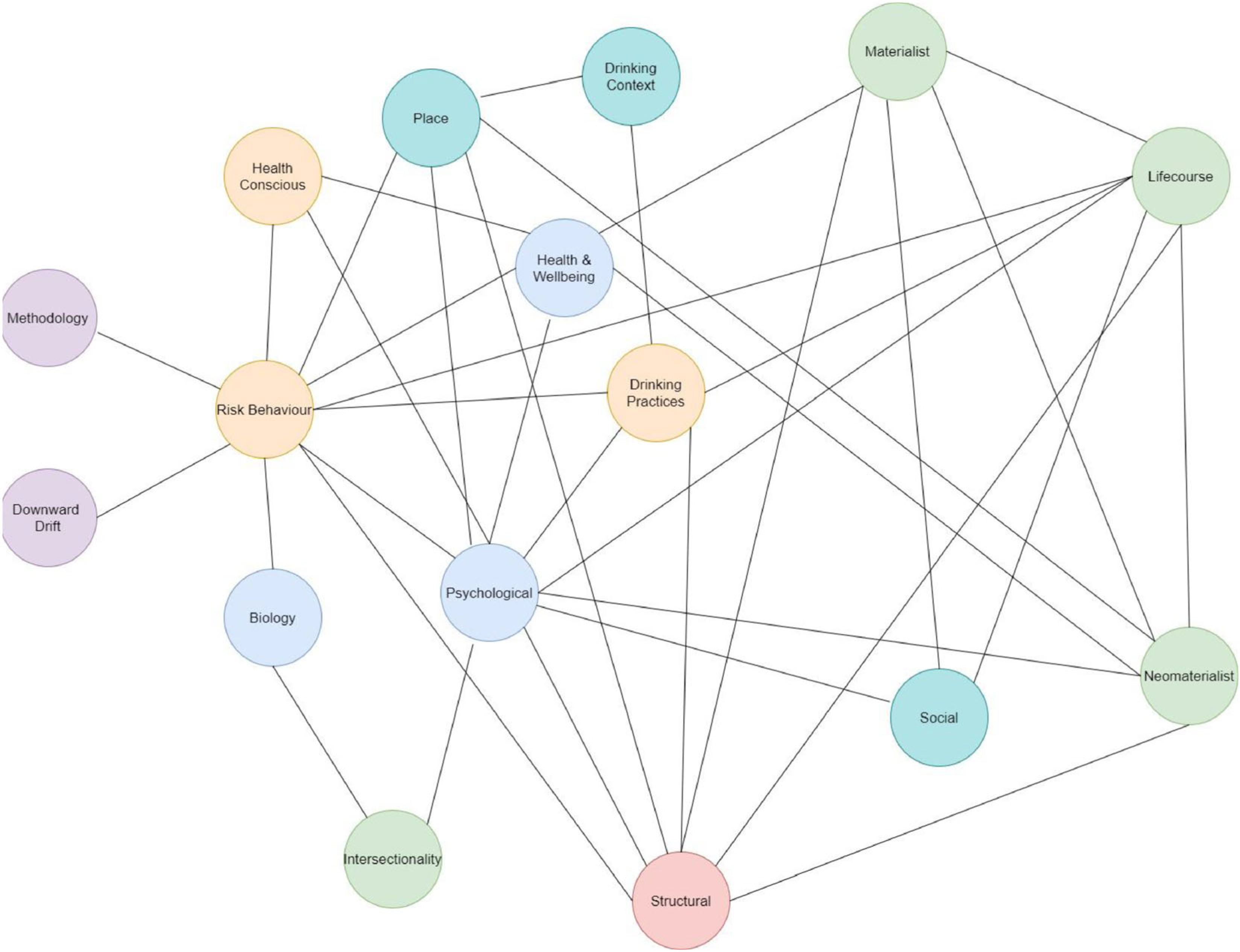
Network diagram illustrating the connections between themes. Domain key: purple = artefactual; orange = life-style; blue = individual; green = disadvantage; turquoise = contextual; red = upstream

Initial coding revealed 41 explanations for the AHP. The explanations were often presented in discussion sections, did not draw upon existing theory and often appeared to be *post-hoc* explanations for findings. Following inductive thematic analysis of the 41 explanations, we identified 16 themes and then grouped these themes into six domains: individual, life-style, contextual, disadvantage, upstream and artefactual. Domains, themes and explanation definitions are shown in Table 3. The number of papers suggesting each theme as an explanation is presented; however, it should be noted that this is a metric of popularity rather than merit. There was no obvious connection between study design or population and the type of explanation given (Table 3). Themes were not mutually exclusive, and authors often combined or indicated interactions between explanations. These relationships are highlighted in a network diagram (Fig. 2).

#### Individual

Individual explanations consisted of processes which take place within individuals that could increase their susceptibility to alcohol-related harm. Themes within this domain included biological (*n* = 7), psychological (*n* = 22) and health and wellbeing (*n* = 19) (Table 3). Explanations within the individual domain were often not amenable to human intervention (e.g. genetic make-up or a pre-existing physical health condition).

Individual explanations for the AHP were only hypothesized and had not been tested within any causal or correlational analyses. In related areas, one author has used the tension reduction model to explain alcohol consumption (the idea that alcohol is consumed as a coping strategy to achieve tension reduction) [33]. There was also some evidence to suggest coping strategies more broadly [8,34], and abstention due to pre-existing health conditions [34,35] differed by SEP. Another paper highlighted that the biological effects of social inequality which leads to higher mortality of lower social classes has been observed in primates [34]. However, given the lack of evidence it is unclear whether these explanations contribute to the AHP.

#### Life-style

The life-style domain focused upon health behaviour of individuals and groups. These were distinct from individual explanations, as they involved an element of choice. Themes were risk behaviour (*n* = 51), drinking practices (*n* = 11) and health-consciousness (*n* = 10) (Table 3). One paper explicitly referred to theories of social practice (the context, how and why of drinking) when discussing how drinking practices at the group level could contribute to the paradox [36]. Another discussed diffusion of innovation theory: the idea that higher SEP groups are faster to adopt new and healthier behaviours [37].

Several papers (*n* = 21) investigated the role of risk behaviour in explaining the AHP. One study highlighted higher rates of hazardous behaviour (e.g. creating a public disturbance or physically abusing someone) among the socio-economically advantaged rather than the disadvantaged [38]. Another study also highlighted that, for young adults, risky alcohol consumption and heavy drinking was more prevalent in the employed compared to the unemployed, while alcohol-related problems were greater for the unemployed [42]. Otherwise, there was evidence to suggest that drinking patterns and clustering of health behaviours may play some role, as several cross-sectional studies highlighted that those of a low SEP tend to engage in heavier drinking patterns and multiple unhealthy behaviours [8,21,22,30,39–41]. Those testing the causal role of risk behaviour (*n* = 13) found that these factors partially attenuate the AHP but could not fully explain excess harm experienced by lower SEP groups [3,4,9,18,43–48]. For example, one record linkage study revealed that when adjusting for alcohol consumption, heavy drinking, BMI and smoking, the hazard ratio for the most deprived group compared to the least deprived was 2.71 [95% confidence interval (CI) = 2.01–3.64] [4]. However, two studies found that controlling for drinking pattern completely accounted for differences in alcohol-related problems in an adult and young adult population [49,50]. In contrast, there was no evidence on the impact of drinking practices or the protective effects of health-consciousness.

#### Contextual

Contextual factors were those in the individual’s immediate environment which may contribute to the AHP. Themes included social (*n* = 20), drinking context (*n* = 11) and place (*n* = 18) (Table 3).

Although widely discussed, contextual explanations lacked empirical testing. One study, using a within- and between-subjects design, found that when individuals live in neighbourhoods with higher levels of poverty they report 5% more negative alcohol consequences compared to when they lived in a wealthier area [credible interval (CR) = 1.05; 95% CI = 1.00, 1.11; *P* = 0.045] and those who, on average, reside in more impoverished areas also report more negative alcohol consequences (CR = 1.27; 95% CI = 1.10, 1.46; *P* = 0.001) [51]. Some studies provided evidence that social factors (e.g. marital status) provide a protective effect [9,52]. However, the limited evidence on other contextual factors, including the relationship between outlet density, consumption and harm, was mixed [53,54].

#### Disadvantage

Explanations in the disadvantage domain tended to focus upon the lived experience of those in poverty and how different facets of this may contribute to the AHP. Themes included intersectionality (*n* = 8), life-course (*n* = 14), material (*n* = 10) and neo-materialist (*n* = 21) (Table 3).

Despite repeatedly appearing in the discussion sections of included papers, only a few explanations associated with disadvantage were empirically tested. Adjusting for material and behavioural factors [45] or cumulative behaviours during the life-course [37] attenuated the relationship between SEP and harm by 18–31% and 38–77%, respectively. There was also evidence that early SEP, disadvantage during adulthood and negative prenatal factors (e.g. maternal heavy drinking) all increased the risk of developing a comorbid mental health and alcohol use disorder, which was not attenuated when controlling for own adolescent drinking [55].

#### Upstream

The upstream domain captured explanations at the macro-level which were hypothesized to have effects on alcohol-related harm. Themes included economic (*n* = 11), socio-political (*n* = 7), alcohol policy (*n* = 5), corporate influence (*n* = 1), employment (*n* = 8), power (*n* = 1) and broad determinants (*n* = 4) (Table 3). These explanations focused upon the structure of society rather than factors associated with belonging to SEP groups. However, the pathways between these societal structures and alcohol harm were not well explained.

None of the included papers attempted to empirically assess whether structural factors can account for the AHP. There was evidence to suggest that economic stressors are more closely associated with mortality in the lowest SEP groups [33,56]. There is also mixed evidence that negative health effects associated with job loss are concentrated in those already at risk due to pre-existing alcohol problems [57], and that SEP overlaps with harmful occupational exposures [43]. However, the extent to which these contribute to the AHP is unknown.

#### Artefactual

Artefactual explanations claimed the AHP was found due to error. Themes included downward drift (*n* = 9) and methodological (*n* = 16) (Table 3).

There was evidence which opposed artefactual explanations for the AHP. Although downward drift was commonly discussed, the only study to test it found that it could not account for the AHP [4]. Record linkage and longitudinal studies also support the existence of the paradox [4,9,37,40,41,45,52,55,57–63], and therefore diminished concerns of under-representation of low-income heavy drinkers in the alcohol consumption data. Another study highlighted that adjusting for alcohol biomarkers only slightly attenuated socio-economic differences in alcohol mortality (1.0–12.1%), suggesting that measurement error is not a probable explanation for the AHP [64]. There was a lack of evidence investigating the impact of often unmeasured factors (e.g. type of cigarette).

#### Relationships between the thematic explanations

The relationships between all themes (colour-coded for domain) are shown in Fig. 2. The connections represent where authors have combined themes within a single explanation. For example, the methodology theme is connected to risk behaviour, as one explanation argues that lower SEP groups drink more than they self-report and their heavy consumption leads to greater harm [60].

It is clear that risk behaviour is central to explanations for the AHP, with the greatest number of connections to other themes (*n* = 10) and links with every other domain (Fig. 2). This is unsurprising, given that health risk behaviours have been the focus of empirical efforts to understand the causes of the AHP.

Other themes, specifically within the upstream and disadvantage domains, were also well connected, possessing connections to four of the five domains. Despite this, they lacked empirical testing.

However, some themes—biological, intersectionality, drinking context and those in the artefactual domain—only had one or two connections. This could reflect the characteristics of the explanation; for example, one of the methodological explanations suggests that, due to the use of self-report measures, research has failed to capture accurate levels of alcohol consumption for low SEP groups: they consume more than they report. Alternatively, the lack of connectivity could reflect value in terms of what researchers think are important explanations for the paradox.

## DISCUSSION

This review examined explanations for the AHP to identify potential pathways and mechanisms which result in differential risk of harm between SEP groups. This is a new approach, and goes beyond previous systematic reviews and meta-analyses which have so far established the existence of the AHP and the contribution of alcohol to this relationship [3,18]. We identified 16 themes within six domains used to explain the AHP. Risk behaviours were the most prevalent explanations. This finding, paired with the dominance of the behavioural paradigm in empirical work, suggests that there has been a reliance upon using risk behaviour to understand the AHP. Evidence found in this review opposed the idea that the AHP was an artefact. There were many other, mainly hypothetical, explanations for the AHP proposed in the literature. This included individual-level mechanisms (e.g. biological or psychological), contextual factors (e.g. place-based factors), the lived experience of disadvantage and upstream structural factors (e.g. the economy and politics). In part, this reflects an awareness that the AHP is complex; there is no simple explanation, and researchers do not view causes in isolation. However, it remains unclear why other re-occurring explanations (e.g. social support or access to health care) have been neglected, while researchers frequently return to risk behaviours. This is particularly puzzling, given that quantitative evidence suggests that risk behaviours only play a partial role [4,47].

There are two potential reasons for this: theoretical and methodological. Study of the AHP is rooted in alcohol epidemiology, which singularly focuses upon the causes and effects of alcohol consumption [65]. More broadly, the field of epidemiology has faced criticism regarding its approach to understand population health. One of the earliest critiques by Krieger points to fundamental errors in developing epidemiological methods rather than theory, with greater weight given to proximal risk factors and a focus upon causes without context [66]. These limitations have led to an emphasis upon individual disease susceptibility and individual-level interventions. Instead, Krieger argues that the eco-social perspective (the idea that biology and biological changes are shaped by the social environment) should be used to understand health [66]. Concerns regarding how causation is viewed in epidemiology have persisted in contemporary public health, with similar criticisms raised more recently [67]. These concerns continue, despite efforts to raise the profile of theories such as the eco-social perspective and calls to adopt pluralist approaches to causality in epidemiology, which stipulate that causation is not a single connection between two things, but the context in which a causal relationship is observed plays a role [67]. Adopting such an approach would change the way alcohol researchers conceptualize and investigate the AHP.

The lack of clear theoretical structuring in epidemiology, which is argued to have led to a focus upon proximal risk factors (e.g. risk behaviours), could also be a symptom of a lack of methods to carry out more complex analyses of distal factors. Possible solutions to this include the use of complex system modelling methods, which have gained traction within public health and are now being implemented in a UK-based project to gain insight into the causal relationships between policy and health-related outcomes [68]. Software architecture has also recently been devised to address how theory can be systematically incorporated into individual-level and agent-based computer simulations to understand health and health behaviours [69]. Applying these computer simulation methods to the AHP could provide the opportunity to shift the empirical focus from risk behaviours to wider determinants, as they can capture complexity and are mechanism-based rather than focused upon testing relationships between variables.

### Strengths and limitations

This is the first review, to our knowledge, to catalogue explanations provided for the AHP across a breadth of literature. In taking a broad approach to literature searching and inclusion criteria it was possible to review work from multiple disciplines employing varied methodologies. This led to the identification of a varied set of explanations. However, it is possible that some explanations are more appropriate, depending upon the study design, population and measure of harm. As the primary aim of this review was to collate and review explanations more generally, we did not conduct an in-depth exploration of this issue. However, upon examination there was no evidence that study design or population influenced which explanations were presented. In terms of measures, we found one clear example of an explanation only applicable when using a subjective measure of alcohol harm—those in low SEP groups who drink may feel their outcomes are worse because their peers are more likely to be abstainers [8]. This issue awaits further examination.

This review was restricted to high-income countries. The results and conclusions are therefore only applicable to this context. Furthermore, most papers focused upon the United Kingdom, which may limit generalizability. This was justified, given substantial differences in alcohol environments. However, given that alcohol is a global issue [1], future research should gain insight into how alcohol affects the disadvantaged in low–middle-income countries to help address the deepening of local and global health inequalities.

Another limitation is that only one reviewer screened and extracted data from the papers. We recruited an independent researcher to re-assess a sample of papers for inclusion and extraction. Cross-checking between the two reviewers demonstrated good reliability.

### Research and policy implications

The lack of explicit theory used to present explanations is a barrier to understanding the causes of the AHP. The development or application of theory may be fundamental to identify the true causal mechanisms which create and sustain the AHP. Several explanations have been proposed which align with the vast literature detailing theories of health inequality more generally. The eco-social perspective, among those more commonly discussed [e.g. the materialist (the link between wealth and resources and health) or political economy perspective (the idea that risk factors for health inequalities are rooted in structures)] [70], are just some examples of health inequality theory which could be applied to understand the AHP.

The AHP is well-evidenced, and behavioural-related explanations play a partial role. However, these explanations fall short in understanding the complex causes of inequalities in alcohol-related harm. There is a current lack of evidence investigating other explanations found in this review, which makes it difficult to suggest potential interventions to mitigate the AHP. Future research should empirically investigate these alternative explanations for the AHP. Computer simulations models offer one potential way of achieving this aim in the short term and for relatively low cost.

Based on the evidence from this review, the key policy implication is that tackling drinking alone will not reduce inequalities in alcohol-related harm. While there is some evidence that improving multiple health behaviours may attenuate the risk of alcohol-related harm, it is critical that policymakers look to policies outside the scope of public health to mitigate the inequality produced by the paradox.

## Conclusions

There are many proposed explanations for the AHP; however, efforts thus far have revolved around risk behaviours as the main cause. Other potentially promising explanations associated within the individual, contextual, disadvantage and upstream domains have remained hypothetical and understudied. Implementation of health inequality theory and complex modelling techniques could provide the opportunity to explore the role of wider determinants in creating and sustaining the AHP.

Additional supporting information may be found online in the Supporting Information section at the end of the article.

## Supplementary Material

sm1 **Table S1** Systematic Search Strategy
**Table S2.1** AXIS Critical Appraisal for included cross-sectional studies.
**Table S2.2** CASP Critical Appraisal for included case–control studies.
**Table S2.3** CASP Critical Appraisal for included cohort studies.
**Table S2.4** CASP Quality Appraisal for included qualitative studies
**Table S2.5** CASP Quality Appraisal for included systematic reviews.

## Figures and Tables

**Table 1:** Population, exposures, comparisons, outcomes and study design criteria for study inclusion.

Criteria	Definition

Population	OECD high-income countries only
Exposures	Alcohol consumption (any measure including both self-report (e.g. quantity/frequency, heavy drinking occasions), biological indicators (e.g. blood alcohol concentration) and aggregate sales data (e.g. per-capita consumption)
Comparisons	Socio-economic position [any measure including area-level deprivation and individual measures (e.g. educational attainment, occupation and income level)]
Outcomes	Alcohol-related harm [any measure which relates to health harms (e.g. morbidity and mortality), clinical diagnosis of alcohol use disorder using ICD codes or DSM-IV manual or negative alcohol-related consequences (e.g. had an accident)]
Study designs	All designs were considered both quantitative and qualitative—including secondary research, intervention studies were excluded

OECD = Office for Economic Cooperation and Development.

**Table 2:** Characteristics of included papers.

Author, year	Country	Study design	Study year	Population	Sample size	Age	Measurement level	Harm measure	SEP measure	Consumption measure	Evidence of the AHP

Alcohol Research UK, 2015 [53]	UK	Report	NA	NA	NA	NA	NA	NA	NA	NA	NA
Backhans *et al.* 2016 [57]	Sweden	Cohort	2002–11	G	15 841	18–84	I	AR hospital, death	ES, E	Last 12 months; drinks/week; binge drinking	Yes
Beard *et al.* 2016 [10]	UK	Cross-sectional	2014–15	G	1700	16+	I	AUDIT-H, AUDIT-D	O, I, E, ES, H	AUDIT-C	Yes
Beilis & Hughes, 2009 [39]	UK	Report	NA	NA	NA	NA	NA	NA	NA	NA	NA
Beilis *et al.* 2016 [21]	UK	Cross-sectional	2013–14	G	6015	18+	I, AG	NA	A	Last 12 months, units/week	NA
Bloomfield, 2020 [71]	Denmark	Commentary	NA	NA	NA	NA	NA	NA	NA	NA	NA
Boyle *et al.* 2014 [72]	Australia	Case–control	2005–07	G	918 (cases), 1021 (controls)	40–79	I, AG	Colorectal cancer	A	g/week	NA
Breakwell *et al.* 2007 [73]	UK	Cross-sectional	1991–2004	G	NA	15+	AG	AR death	A	Units/week	Yes
Brown *et al.* 2014 [33]	USA	Cross-sectional	2010–11	G	663	19–91	I	Somatic complaints	E	Drinks/month	NA
Chick, 1998 [34]	UK	Review	NA	NA	NA	NA	NA	NA	NA	NA	NA
Collins, 2016 [16]	USA	Review	NA	NA	28 studies	NA	NA	NA	NA	NA	NA
Connor *et al.* 2010 [54]	New Zealand	Cross-sectional	2006–07	G	1770	18–70	I, AG	Negative AR consequences	E, A	Drinking days last 12 months, drinks/occasion, binge drinking	NA
Conway *et al.* 2015 [74]	EU, Americas	Case–control	1988–2007	G	23 964 cases, 31, 954 controls	NR	I	Head and neck cancer	E, I	Drinker status, drinks/day	Yes
Degerud *et al.* 2018 [40]	Norway	Cohort	1960–2011	G	207 394	NR	I	Cardiovascular disease, ischaemic heart disease, cerebrovascular and all-cause mortality	H, I, E	g/day, heavy drinking episodes	Yes
Evans-Polce *et al.* 2016 [35]	UK	Cohort	1958–2006	G	11 469	7–55	I	All-cause mortality	PI, H, O	Units/week	NA
Fair Foundation, 2015 [75]	Australia	Report	NA	NA	NA	NA	NA	NA	NA	NA	NA
Fillmore *et al.* 1998 [76]	USA, Sweden	Meta-analysis	1964–82	NA	31 studies	16+	I	All-cause mortality	E, ES, I	Drinks/occasion, occasions/month, drinks/month	Yes
Gartner *et al.* 2019 [47]	Wales	Record-linkage	2013–16	G	11 038	16+	I	AR hospital	A, SC, E, ES, H	Units/heaviest drinking day, last 12 months	Yes
Hall, 2017 [77]	UK	Commentary	NA	NA	NA	NA	NA	NA	NA	NA	NA
Hart, 2015 [78]	Australia	Qualitative	NA	Young adults	NA	18–24	I	NA	NA	NA	NA
Herttua *et al.* 2007 [56]	Finland	Cohort	1985–2003	G	70.1 million	15+	I, AG	AR death	E	Litres/capita	NA
Huckle *et al.* 2010 [49]	New Zealand	Cross-sectional	1995, 2000, 2004	G	3848, 4295, 5477	18–65	I	Negative AR consequences	E, I, O	Litres/year	Yes
Jonas *et al.* 1999 [79]	Australia	Cross-sectional	1995–96	G	NR	NA	AG	AR hospital	ES, O, H, I, MV	Litres/capita	NA
Jones *et al.* 2015 [18]	EU, Americas	Systematic review	2012	NA	31 studies	NA	I, AG	AR morbidity, death	E, O, I, A, ES, H, OM	g/year, g/day, drinks/week, units/week, drinks/day, drinking status, ml/day, days drank/week, glasses/day, binge drinking, years vodka consumption, drinks/last 12 months	Yes
Karriker-Jaffe *et al.* 2012 [80]	USA	Cross-sectional	2000, 2005	G	7613, 6919	18+	I, AG	Negative AR consequences, AD	A	Drinks/last 12 months, Heavy drinking	Yes
Karriker-Jaffe *et al.* 2013 [81]	USA	Cross-sectional	2000, 2005	G	7613, 6919	18+	I, AG	Negative AR consequences	A	Drinks/last 12 months, Heavy drinking	Yes
Katikireddi *et al.* 2017 (a) [4]	UK	Record-linkage	1995–2012	G	50 236	M = 48	I, AG	AR hospital, death and prescription	E, A, O, I	Units/week, binge drinking	Yes
Katikireddi *et al.* 2017 (b) [82]	UK	Commentary	NA	NA	NA	NA	NA	NA	NA	NA	NA
Kuendig *et al.* 2008 [83]	EU	Cross-sectional	1997–2002	G	NA	25–60	I	Negative AR consequences	E, ES	g/day, binge drinking	Yes
Lawder *et al.* 2011 [63]	UK	Cohort	1998–2008	G	8305	M = 47	I, AG	AR hospital	ES, B, A	Units/week	Yes
Lewer *et al.* 2016 [22]	UK	Cross-sectional	2008–13	G	51 498	18+	I, AG	NA	I, E, ES, A	Heavy episodic drinking, Heavy weekly drinking	NA
Livingston, 2014 [38]	Australia	Cross-sectional	2010	G	21 452	12+	I, AG	NA	A, I	Drinks/year, risky drinking	NA
Lundin *et al.* 2012 [58]	Sweden	Cohort	1969–91	MC	37 798	18+	I	AR hospital	PI, O, E, I	Risky alcohol use	Yes
Major *et al.* 2014 [59]	USA	Cohort	1995–2006	G	4 814 247	M = 63	I, AG	Hepatocellular carcinoma incidence, chronic liver disease mortality	A	Drinks/day	Yes
Makela & Paljarvi, 2007 [9]	Finland	Cohort	1969–2000	G	6406	25–69	I	AR hospital, death	O	Cl/year	Yes
Makela, 2008 [84]	Finland	Commentary	NA	NA	NA	NA	NA	NA	NA	NA	NA
Marmot, 2001 [85]	UK	Commentary	NA	NA	NA	NA	NA	NA	NA	NA	NA
Mayor, 2016 [86]	UK	Commentary	NA	NA	NA	NA	NA	NA	NA	NA	NA
McDonald *et al.* 2008 [60]	UK	Record-linkage	1995–2005	G	23 183	30+	I, AG	AR discharge diagnosis	A	Units/week	Yes
Meier *et al.* 2017 [36]	UK	Discussion	NA	NA	NA	NA	NA	NA	NA	NA	NA
Menvielle *et al.* 2004 [43]	France	Case–control	1989, 1991	MP	504 cases, 242 controls	< 50–70	I	Laryngeal or hypopharyngeal cancer	E, O, OM	Glasses/day	Yes
MESAS, 2016 [87]	UK	Report	NA	NA	NA	NA	NA	NA	NA	NA	NA
Moller *et al.* 2019 [48]	Denmark	Cross-sectional	2014	Young adults	70 566	M = 17.9	I	Negative alcohol consequences	PI	Standard drinks/week	Yes
Mulia & Karriker-Jaffe, 2012 [88]	USA	Record-linkage	2000, 05	G	13 231	24+	I, AG	Negative alcohol consequences, AD	E, A	Drinking status, risky drinking, monthly drunkenness	NA
Mulia & Zemore, 2012 [89]	USA	Cross-sectional	2005	G	4080	18+	I	AD	Poverty status	Frequency of drunkenness in the last year	NA
Nielsen *et al.* 2004 [90]	Denmark	Cohort	1976–2001	G	14 223	20+	I	All-cause mortality	E, I	Frequency of types	NA
Norstrom & Landberg, 2020 [91]	Sweden	Cohort	1994–2017	G	NA	NA	AG	Alcohol-specific mortality, violent deaths	E	Per-capita consumption	Yes
Norstrom & Romelsjo, 1999 [30]	Sweden	Cross-sectional	1990, 1991–95	M	2817	20–64	I	AR death	O	Litres/year	No
Nweze *et al.* 2016 [92]	USA	Cross-sectional	2013	P	738	15–70	I	AR hospital	ES, IN	BAC	NA
Parkman *et al.* 2017 [93]	UK	Qualitative	2015	P	30	16+	I	AR hospital	E, H, ES	Current and previous use	NA
Pena *et al.* (2020) [64]	Finland	Eight cohort studies	1978–2016	G	52 164	25+	I	AR death	I, E	g/week, alcohol biomarkers	Yes
Pena *et al.* (2021) [46]	Finland	Eight cohort studies	1978–2016	G	53 632	25+	I	AR death	I, E	g/week	Yes
Probst *et al.* 2020 [3]	Canada	Systematic review/meta-analysis	2020	NA	10 studies	NA	NA	NA	NA	NA	Yes
Public Health Wales, 2014 [94]	UK	Report	NA	NA	NA	NA	NA	NA	NA	NA	NA
Rehm & Probst, 2018 [95]	Canada	Discussion	NA	NA	NA	NA	NA	NA	NA	NA	NA
Rhew *et al.* 2020 [51]	USA	Cohort	NA	Young adults	746	18–23	I	Negative alcohol consequences	PI	Standard drinks/week	NA
Roberts *et al.* 2008 [32]	UK	Record-linkage	1998–2003	P	52 096	< 35–> 75	I, AG	Pancreatitis incidence, death	A	Binge drinking	No
Roberts *et al.* 2013 [31]	UK	Record-linkage	1999–2010	P	19 196	< 35–> 75	I, AG	Pancreatitis incidence, death	A	Units/day in the previous week	No
Roche *et al.* 2015 [96]	Australia	Review	NA	NA	138 studies	NA	NA	NA	NA	NA	NA
Romelsjo & Lundberg, 1996 [97]	Sweden	Cross-sectional	1967–93	G	NR	25–64	I	AR hospital, deaths	O	g/day	Yes
Sadler *et al.* 2016 [98]	UK	Cross-sectional	2010–13	P	9.6 million HES alcohol admissions	18+	AG	AR hospital	A	NA	NA
Salom *et al.* 2014 [55]	Australia	Cohort	1981–2002	Young adults	2399	0–21	I	Mental health, AD	ES, PI, PES	Drinks/occasion	Yes
Sargent, 1989 [61]	Australia	Discussion	NA	NA	NA	NA	NA	NA	NA	NA	NA
Shaper *et al.* 1988 [62]	UK	Cohort	1978–87	M	7735	40–59	I	All-cause mortality	O	Units/week	Yes
Singh & Hoyert, 2000 [52]	USA	Cohort	1979–89, 1990–92	G	370 500	25+	I, AG	Cirrhosis and chronic liver disease mortality	ES, E, PI, O	Per-capita consumption	Yes
Skogen *et al.* 2019 [99]	Norway	Cross-sectional	NA	G	4311	16–72	I	AUDIT	O, I, ES	AUDIT-C	NA
Smith & Foster, 2014 [14]	UK	Report	NA	NA	NA	NA	NA	NA	NA	NA	NA
Stanford-Moore *et al.* 2018 [44]	USA	Case–control	2002–06	P	1153 cases, 1267 controls	20–80	I	Squamous cell carcinoma of the head and neck	I, E, IN	Drinking status, years drank, g/lifetime	Yes
Stewart *et al.* 2017 [41]	UK	Cohort	2000–14	Adults with LTC	95 991	18+	I, AG	All-cause mortality	A	Drinking status, units/week	Yes
Syden *et al.* 2017 [45]	Sweden	Cohort	2002–11	G	17 440	25–64	I	AR hospital, death	O	g/week, Heavy drinking	Yes
Thern *et al.* 2019 [42]	Sweden	Cohort	2013–14	Young adults	1005	17–29	I	AUD	ES	Weekly binge drinking	Yes
Thor *et al.* 2019 [50]	Sweden	Cross-sectional	2015–16	Young adults	6153	17–18	I	Negative alcohol consequences	PI, A, academic orientation	Binge drinking	Yes, for 2/3 SEP measures
Trias-Llimos *et al.* 2020 [100]	Europe	Cross-sectional, cohort	2011–15	G	159 132 person – years at risk	50–85	I	All-cause mortality	E	AUDIT-C	Yes
Van Oers *et al.* 1999 [8]	the Netherlands	Cross-sectional	1994	G	3537	16–69	I	Negative alcohol consequences	E	Type, days/month, glasses/occasion	Yes
Whitley *et al.* 2014 [37]	UK	Cohort	1990–2008	G	C1 = 1444, C2 = 1550	35+	I	All-cause mortality	O, I, E	Units/week	Yes
WHO, 2014 (a) [101]	Global	Report	NA	NA	NA	NA	NA	NA	NA	NA	NA
WHO, 2014 (b) [1]	Global	Report	NA	NA	NA	NA	NA	NA	NA	NA	NA
WHO, 2018 [102]	Global	Report	NA	NA	NA	NA	NA	NA	NA	NA	NA
Wood & Beilis, 2015 [103]	EU	Report	NA	NA	NA	NA	NA	NA	NA	NA	NA

AUDIT = Alcohol Use Disorders Identification Test; NR = not reported; NA = not applicable; G = general population; MC = military conscripts; P = patient; MP = male patients; M = males; LTC = long-term conditions; I = individual; AG = aggregate; AR = alcohol-related; AD = alcohol dependence; AUD = alcohol use disorder; ES = employment status; E = education; O = occupational social grade; I = income; H = home ownership; A = measure of area-level deprivation; OM = occupational mobility; IN = insurance; PI = parental indicators; PES = partner employment status; MV = motor vehicles; B = benefits; SC = social class; SEP = socio-economic position; G = grams; CL = centilitres; BAC = blood alcohol content.

**Table 3:** Thematic table of explanations for the AHP extracted from included papers with information on type of study design and population.

Domain	Theme	Explanation	Definition	Study design	Population

Individual	Biological	Biological characteristics [3,34,40]	SEP groups have a different biological or genetic make-up related to ethnicity or due to experiencing inequality which leaves them more susceptible to harm	Systematic review, discussion paper, cohort	General population
		Behavioural-related alterations [21,40,46,85,102]	Engaging in multiple risk behaviours has a biological impact: (i) nutritional deficiencies and metabolic consequences which alter protein and vitamin absorption, (ii) an adverse effect on the immune system and (iii) they interact with live enzymes, all leading to greater risk of disease (e.g. liver disease) and harm	Cross-sectional, cohort, commentary, report	General population
	Psychological	Stress [3,4,34,46,52,76,81]	Low SEP groups experience more psychological stress and a greater number of stressful events: (e.g. marital breakdown, dangerous environment, immigrant status, unemployment and living in poverty). This is thought to reduce resilience to disease	Systematic review, cohort, discussion paper, meta-analysis, cross-sectional	General population
		Coping [8,30,33,34,42,52,56,58,78,80,88,89,93,98]	Differences in coping strategies: low SEP groups use alcohol as a coping strategy which can lead to alcohol dependence. They are also more likely to use resigned acceptance as a coping strategy and are less likely to use cognitive avoidance and emotional discharge which independently negatively impact wellbeing	Cross-sectional, discussion paper, cohort, qualitative	General population, men, young people, military conscripts, patient
		Stereotypes/stigma [61,80,81,88,89,96]	Lower SEP groups experience more labelling and discrediting which leads to social rejection and exclusion. This could result in a self-fulfilling prophecy, whereby members of that group enact the behaviours they are expected to possess. This could also increase group and individual tensions which find an outlet via harmful drinking. This may also lead to fewer social resources, increasing psychological vulnerability	Discussion paper, cross-sectional, cohort, review	General population
		Attribution [8,83]	There are a higher number of abstainers in low SEP groups, therefore the alcohol problems faced by those who do drink in this group may seem worse by comparison. This only holds true for subjective measures of alcohol-related harm	Cross-sectional	General population
	Health and wellbeing	Physical health [8,9,32,34,35,41,47,62,63,74,76,93,94,96,98]	There is a higher prevalence of pre-existing physical health conditions, poorer general health, multi-morbidities or being overweight/obese in low SEP groups which could explain disproportionate effects of alcohol	Cross-sectional, cohort, review, case–control, meta-analysis, qualitative, report	General population, patient, men, adults with long-term conditions
		Mental health [8,22,34,41,47,48,50,53,63,76,93,96,98]	Low SEP individuals tend to be more psychologically vulnerable and have a greater prevalence of pre-existing mental health conditions, mental distress, or psychological symptoms (e.g. nervousness, irritability, helplessness, loneliness) which could exacerbate the effects of alcohol. There is also an independent association between poor wellbeing and worse health outcomes	Cross-sectional, review, cohort, report, meta-analysis, qualitative	General population, patient, adults with long-term conditions, young adults
Life-style	Risk behaviour	Drinking patterns [1,3,4,8,10,14,18,21,22,30,32,34,39,41,45–47,49,51–54,59,60,62–64,71,73,79,81,83,85,86,91,94,95,98–100,103]	Although overall or average alcohol consumption may be similar, or lower for low SEP groups, they consume greater quantities of alcohol per drinking occasion	report, systematic review, meta-analysis, cross-sectional, cohort, review, commentary, discussion paper	General population, men, patient, young adults
		Clustering of health behaviours [3,4,10,18,21,22,37,40–44,46,47,53,59,63,64,72,74,76,81,90,95,103]	Those in low SEP groups engage in multiple health risk behaviours for example smoking, poor diet, a lack of exercise and concurrent drug use which exacerbate the impact of alcohol	Systematic review, meta-analysis, cross-sectional, cohort, case–control, report, discussion paper	General population, adults with long-term conditions, young adults, male patients, patient
		Type of beverage [4,10,18,21,32,47,86,90,93,102]	Beers, ciders and spirits are more commonly consumed by low SEP, while wine is often associated with higher SEP. The quality and price of alcohol consumed may impact harm outcomes	Cohort, systematic review, meta-analysis, cross-sectional, commentary, qualitative, report	General population, patient
		Drinking history/future drinking [4,21,22,60,62,77,103]	Drinking is temporal and may change throughout the life-course. Although those of low SEP may have reduced consumption upon measurement, increased susceptibility to harm could be due to previous drinking. There are several reasons why people may reduce consumption (e.g. developing an illness). This explanation was extended to an increase in consumption in the future, as some studies only measure consumption at baseline and outcomes in following years	Cohort, cross-sectional, commentary, report	General population, men
	Drinking practices	Norms [51,53,75,78,85,87,92,96,101]	Group and neighbourhood norms including drinking pattern, expected volume, how to drink certain beverages (e.g. shot a spirit) and norms around the permissibility of excessive alcohol use differs by SEP	Cohort, report, qualitative, review	Young adults, patient
		Culture [75,78,88,96]	Drinking culture attached to certain places of employment or neighbourhoods may lead to poorer health and difficulties maintaining employment, which could then exacerbate stress and increase consumption	Report, qualitative, cohort, review	Young adults, general population
	Health-consciousness	Health literacy [21,37,77,93,94,97]	Engagement with health promotion campaigns and preventative services. It was proposed that low SEP may not make use of available services or are slower to access these services	Cross-sectional, cohort, commentary, qualitative, report	General population, patient, men
		Healthy behaviours [9,34,37,76,85]	Those of a high SEP adopt healthy behaviours (e.g. good diet and exercise) which may protect against negative impacts of drinking	Cohort, review, meta-analysis, commentary	General population
Contextual	Social	Social support [9,18,34,35,45,51–53,55,73,75,76,89,93,96,101–103]	Social support may buffer the negative impacts of alcohol consumption. Those of high SEP have a wider ‘social margin’ which insulates them from the negative consequences of their actions while low SEP lack social support and are often socially isolated	Systematic review, meta-analysis, report, cross-sectional, review, cohort	General population, young adults
		Social exclusion [1,75,76,88,96]	The marginalization of low SEP groups is greater due to several factors including a higher number of abstainers, stigmatization that comes with having an alcohol use disorder and intersections between multiple minority status (e.g. ethnic, refugee, homeless and LGBT+)	Report, meta-analysis, cohort, review	General population
		Peer influence [9,53,75,96,101,102]	Negative influence from peers and family in low SEP groups may impact harm outcomes. There is evidence that men of high SEP are more likely to be married and therefore long-term partners may be an important agent of social control for excessive drinking. Not only would a partner provide social control but also additional financial support *via* combined income and this influence was extended to others in their social network	Cohort, report, review	General population
	Drinking context	Dangerous environment [1,9,18,36,48–50,78,84,98,102]	Low SEP are more likely to drink in dangerous environments with a lack of policing and safety, which may lead to a higher risk of violence, police encounters and unintentional injury	Report, systematic review, meta-analysis, cohort, discussion paper, cross-sectional, qualitative, commentary	General population, young adults, patient
		Exposure [102]	Drinking in public places is common among the most deprived groups (e.g. the homeless). This leaves them exposed to certain infectious diseases (e.g. TB and HIV) which may compound harm	Report	NA
	Place	Neighbourhood deprivation [10,18,44,46,50,53,79,81,84,89,102]	A lack of resources, treatment facilities or preventative/educational programs, an increased police presence, neighbourhood disorder, low educational ethos and a lack of community institution negatively impact harm outcomes	Systematic review, meta-analysis, cross-sectional, report, case–control, cohort, commentary	General population, patient, young adults
		Alcohol outlet/advertising density [3,53,54,59,71,75,81,87,89,96,102]	Increased outlet density has an impact on patterns of drinking and harmful consequences. The density of alcohol advertising in deprived areas was also considered to potentially influence the excess harm experienced by those of a low SEP	Systematic review, report, cross-sectional, cohort, commentary, review	General population
Disadvantage	Intersectionality	Multiple minorities [44,52,76,80,81,92,96,101]	The impact of belonging to multiple minority groups (e.g. SEP, race, gender, and sexuality), and how experiencing multiple aspects of disadvantage may amplify inequalities in alcohol-related harm	Case–control, cohort, meta-analysis, cross-sectional, review, report	Patient, general population
	Life-course	Cumulative effects [9,37,44,52,55,58,74,84,96,102]	The accumulation of negative/stressful life events over time or additive effects of prolonged risky health behaviours which negatively impacts health and potentially employment itself	Cohort, case–control, commentary, review, report	General population, patient, military conscripts
		Early risk factors [9,50,55,58,75,96,103]	The experience of ACE’s in childhood, childhood household dysfunction and a disadvantaged start in life (including prenatal factors) perpetuates a vicious cycle of poverty and poor health which impacts on social participation, wellbeing, their ability to cope and access to available support or treatment	Cohort, cross-sectional, report, review	General population, young adults, military conscripts
		Family influence [55,81,102]	Limited family income restricts material resources and creates stress given the inability to meet basic needs. Family history of alcohol problems could impact alcohol consumption and health in later life. Parental education is shown to negatively impact on health literacy and children’s employment aspirations, opportunities, and adulthood income	Cohort, cross-sectional, report	General population
	Material	Material resources [1,4,14,49,51,55,73,74,93,96]	A lack of resources could negatively impact on harm due to the inability to protect themselves from the experience of a problem or stressful life event and could exacerbate poor health through poor housing conditions, homelessness, and unemployment	Report, cohort, cross-sectional, case–control, qualitative, review	General population, young adults, patient
	Neo-materialist	Access, quality and barriers [1,3,10,14,18,21,38,44,46,52,53,55,64,75,76,87,93,96,98,102,103]	Depending on geographical distribution, services in disadvantaged areas may be fewer and more difficult to access or of a lower quality. Low SEP groups face several potential barriers when attempting to access health-care including cost, transport, availability (in terms of opening hours), mobility issues and stigma which may deter them from using services. Dependent on country there were additional considerations for example the cost of health insurance	Report, systematic review, cohort, meta-analysis, qualitative, review, cross-sectional, case–control	General population, patient
Upstream	Structural	Economic [1,16,33,45,53,56,75,87,96,97,102]	Trickle-down effects of the economy were thought to contribute to excess harm. Economic stressors (e.g. economic downturns or recession) are more closely associated with morality in the lowest SEP groups. Gross national income and changes in minimum or disposable income has increased the buying power of low SEP groups, which has led to an equalization of alcohol consumption	Report, review, cross-sectional, cohort	General population
		Socio-political [38,44,46,75,80,84,96]	The attitudes and decision making of residents and policymakers. Politicians focusing on individual behaviours rather than tackling the social determinants of health which increases inequalities. Political context is extremely important, as countries with poor minimum living standards, limited public investment in social goods (particularly in deprived areas) and worse social system responses are likely to worsen health outcomes for low SEP groups	Cross-sectional, case–control, cohort, report, commentary, review	General population, patient
		Alcohol policy [41,61,75,87,96]	The mutually beneficial economic relationship between the state and the alcohol industry shapes policy decisions. Although it is hoped that this is counterbalanced by ‘helping professions’ it is also in their interest to continue the expansion of treatment and this is deflected by each entity casting blame on the another. Additionally, a lack of policy that aims to reduce harmful consumption, alcohol availability, pricing and promotion, and global market liberalization (changes in affordability), production, importation, distribution, and pricing of alcohol were hypothesized to contribute to the AHP	Cohort, discussion paper, report, review	Adults with long-term conditions
		Corporate influence [61]	The alcohol industry funds alcohol research which may misinform policy decision-making. Privately owned media was also argued to play a role via diffusing true or false information	Discussion paper	NA
		Employment [9,14,43,52,74,75,81,96]	There were several mechanisms through which employment could worsen alcohol-related harms for low SEP groups. This included the working conditions or occupational exposures faced by low SEP individuals. Job type, low wages and inflexible employment, and job alienation, stress and low satisfaction are all thought to negatively impact harm outcomes. Those from more deprived backgrounds with insecure employment may also be less able to take time off work when they become ill, compounding the problem. This contrasts with the idea that high SEP individuals may get more support from their employers, whereby employers are more willing to invest energy in solving their alcohol problems. Relatedly issues of unemployment were also discussed including the issue of receiving additional help of benefits related to a long-term condition or disability which may discourage some people from getting better as they would lose this additional help as a result	Cohort, report, case–control, cross-sectional, review	General population, male patients
		Power [61]	Dominant groups in society may suppress subordinate groups via different means (e.g. variable wages, segmented social status), therefore fragmenting groups. These subgroups would then experience greater discrimination and stigma, while the status quo is maintained by the dominant groups having individualistic beliefs. This coupled with social control: the idea that the most powerful individuals have an interest in subordinate groups adopting deviant or socially problematic behaviour which in turn is defined by the powerful, facilitates a ‘revolving door’ system by which the same individuals pass through a multitude of institutions including hospitals, jails, and clinics	Discussion paper	NA
		Broad determinants [45,46,85,90]	Other broad factors, such as social and commercial determinants of health, are the causal factors associated with low SEP which may explain the AHP	Cohort, commentary	General population
Artefact	Downward drift	Reverse causation [1,4,21,45,53,57,74,81,85]	Heavier drinkers are more likely to lose their job or move to deprived areas due to their heavy drinking. The existence of an alcohol problem is the driving force behind low SEP, rather than low SEP having an independent association with increased harm	Report, cross-sectional, cohort, report, case–control, commentary	General population
	Methodological	Under-reporting/measurement error [3,14,42,44,47,59,60,64,71,74,90,94,103]	The use of self-report measures allows the opportunity for response bias and memory limitation to impact the results. Measures which rely on binge drinking beyond a threshold instead of individual units is not accurate at capturing differences in the proportions of non-drinkers between SEP groups	Systematic review, meta-analysis, report, cohort, case–control, cross-sectional, commentary	Young adults, patient, general population
		Unmeasured factors [44]	Not all confounders are measured. For example, the way cigarette smoke is inhaled or the type of cigarette could have an impact on harm	Case–control	Patient
		Study Design [46]	Need to use more longitudinal data when investigating the AHP particularly to account for time-dependent effects	Cohort	General population
		Under-representation [3,14,21,85,94]	The heaviest drinkers in deprived areas are often under-represented in studies. This is a potential confounder for cross-sectional studies using aggregate data, as once the heaviest drinkers are accounted for higher rates of harm are no longer paradoxical	Systematic review, meta-analysis, report, commentary	NA

SEP = socio-economic position; LGBT = lesbian, gay, bisexual, transgender; NA = not applicable; TB = tuberculosis.
