# Supplementary material for: Causal mechanisms proposed for the alcohol harm paradox—a systematic review
Source: Addiction. Author manuscript; Available in PMC 2023 Jan 1. (PMC8595457; doi:10.1111/add.15567)
Supplement: sm1 — Table S1 Systematic Search Strategy Table S2.1 AXIS Critical Appraisal for included cross-sectional studies. Table S2.2 CASP Critical Appraisal for included case–control studies. Table S2.3 CASP Critical Appraisal for included cohort studies. Table S2.4 CASP Quality Appraisal for included qualitative studies Table S2.5 CASP Quality Appraisal for included systematic reviews. [file NIHMS1705086-supplement-sm1.docx]

**Supplementary Materials**

**Table S1**: Systematic Search Strategy

| Concept | Search terms |  |  |  |  |
| --- | --- | --- | --- | --- | --- |
| Alcohol  (.mp.) (MEDLINE & Embase) | Alcohol* adj3 drink* | Heavy adj3 drink* | Binge drink* | *alcohol consumption/ or *binge drinking/ or *heavy drinking | Alcohol*.ti. |
| Alcohol (PsychInfo) | Alcohol* adj3 drink* (.mp.) | Alcohol drinking patterns/ | Heavy adj3 drink* (.mp.) | *alcohol consumption/ OR *binge drinking/ OR *heavy drinking/ | Alcohol*.ti. |
| Health Inequalities (MEDLINE & Embase) | Health Status Disparities/ or exp Socioeconomic Factors/ | Health adj2 inequalit* (.mp.) | Socioeconomic or socio-economic (.mp.) |  |  |
| Health Inequalities (PsychInfo) | Health status disparities (.mp.) | Socioeconomic status/ | Health adj2 inequalit* (.mp.) |  |  |
| Socioeconomic Status (MEDLINE & Embase) | Disadvantage* OR inequit* OR inequal* OR poverty OR low income OR unemploy* OR employ | High income OR deprived OR social class OR upper class OR middle class OR working class | Deprivation (.mp.) |  |  |
| Socioeconomic Status (PsychInfo) | Disadvantage* OR inequit* OR inequal* OR poverty OR low income OR unemploy* OR employ (.mp.) | High income OR deprived OR social class OR upper class OR middle class OR working class (.mp.) | Deprivation (.mp.) | *social class/ OR *socioeconomic status/ |  |
| Exclusions for: MEDLINE & Embase | Therapeutics/ OR psychotherapy/ OR intervention.ti. OR brief intervention.ab. OR effectiveness.ti. | (Brain OR bacter* OR pathogen* OR methyl* OR memor* OR cortex OR neur* OR temporal).ti. | (Africa* OR chin* OR india* OR Russia* OR thai* OR vietn* OR Uganda OR brazil OR Nepal).ti. | Addiction.ti. OR rehabilitation.mp. OR psych*.ti. OR rats.mp. OR vehicle.mp. |  |
| Exclusions for: PsycInfo | (Addiction OR rehabilitation OR alcoholi*).ti. | Therapeutics/ OR psychotherapy/ OR intervention.ti. OR brief intervention.ab. OR effectiveness.ti. |  |  |  |

**S2:** Critical Appraisal

Quality Assessment

Overall, the quality of included papers was assessed as good. The key quality concern was non-response bias in cross-sectional studies. Many used secondary data and therefore did not report response rates (e.g. (18,23)) while others reported response rates but did not take measures to address potential biases (e.g. data was not weighted and there were no attempts to contact or categorise non-responders) (17,24,25) (see Tables S2 in supplementary material for full details). Another limitation was studies mainly used self-report measures of consumption and SEP. However, these were often established and validated measures. A focus on physical health harms as an objective outcome measure was a strength of included studies.

NB: Numbers in the top row represent question numbers from respective checklists.

Key: ✓ = meets criteria, DR = do not report, N/A = not applicable, ~ = partially meets criteria, X = does not meet criteria

**Table S2.1:** AXIS Critical Appraisal for included cross-sectional studies.

|  | 1. | 2. | 3. | 4. | 5. | 6. | 7. | 8. | 9. | 10. | 11. | 12. | 13. | 14. | 15. | 16. | 17. | 18. | 19. | 20. |
| --- | --- | --- | --- | --- | --- | --- | --- | --- | --- | --- | --- | --- | --- | --- | --- | --- | --- | --- | --- | --- |
| (1) | ✓ | ✓ | ✓ | ✓ | ✓ | ✓ | DR | ✓ | ✓ | ✓ | ✓ | ✓ | DR | N/A | ✓ | ✓ | ✓ | ✓ | ✓ | ✓ |
| (2) | ✓ | ✓ | ✓ | ✓ | ✓ | ✓ | X | ✓ | ✓ | ✓ | ✓ | ✓ | X 23.3%RR | X | N/A | ✓ | ✓ | ✓ | ✓ | ✓ |
| (3) | ✓ | ✓ | ✓ | ✓ | ✓ | ✓ | N/A | ✓ | ✓ | ✓ | ✓ | ✓ | N/A | N/A | N/A | ✓ | ✓ | X | ✓ | ✓ |
| (4) | ✓ | ✓ | ✓ | ✓ | ✓ | ✓ | X | ✓ | ✓ | ✓ | ✓ | ✓ | X (25.5%RR, 65.8% of those then returned the questionnaire) | X | ✓ | ✓ | ✓ | ✓ | DR | ✓ |
| (5) | ✓ | ✓ | ✓ | ✓ | ✓ | ✓ | ✓ | ✓ | ~ | ✓ | ✓ | ✓ | ✓ | X | DR | ✓ | ✓ | ✓ | ✓ | ✓ |
| (6) | ✓ | ✓ | ✓ | ✓ | ✓ | ✓ | N/A | ✓ | ✓ | ✓ | ✓ | ✓ | ✓ | N/A | N/A | ✓ | ✓ | X | ✓ | ✓ |
| (7) | ✓ | ✓ | ✓ | ✓ | ✓ | ✓ | N/A | ✓ | ✓ | ✓ | ✓ | ✓ | ✓ | N/A | N/A | ✓ | ✓ | ✓ | ✓ | ✓ |
| (8) | ✓ | ✓ | ✓ | ✓ | ✓ | ✓ | ✓ | ✓ | ✓ | ✓ | ✓ | ✓ | ✓ | N/A | N/A | ✓ | ✓ | ✓ | ✓ | ✓ |
| (9) | ✓ | ✓ | ✓ | ✓ | ✓ | ✓ | N/A | ✓ | ✓ | ✓ | ✓ | ✓ | N/A | N/A | N/A | ✓ | ✓ | ✓ | ✓ | ✓ |
| (10) | ✓ | ✓ | ✓ | ✓ | ✓ | ✓ | N/A | ✓ | ✓ | ✓ | ✓ | ✓ | ✓ | N/A | N/A | ✓ | ✓ | ✓ | DR | ✓ |
| (11) | ✓ | ✓ | ✓ | ✓ | ✓ | ✓ | N/A | ✓ | ✓ | DR | ✓ | ✓ | N/A | N/A | N/A | ✓ | ✓ | X | DR | ✓ |
| (12) | ✓ | ✓ | ✓ | ✓ | ✓ | ✓ | N/A | ✓ | ✓ | ✓ | ✓ | ✓ | N/A | N/A | N/A | ✓ | ✓ | ✓ | X | ✓ |
| (13) | ✓ | ✓ | ✓ | ✓ | ✓ | ✓ | ✓ | ✓ | ✓ | ✓ | ✓ | ✓ | ✓ | N/A | N/A | ✓ | ✓ | ✓ | DR | ✓ |
| (14) | ✓ | ✓ | ✓ | ✓ | ✓ | ✓ | X | ✓ | ✓ | ✓ | ✓ | ✓ | ~ | N/A | N/A | ✓ | ✓ | ✓ | ✓ | ✓ |
| (15) | ✓ | ✓ | ✓ | ✓ | ✓ | ✓ | N/A | ✓ | ✓ | ✓ | ✓ | ✓ | N/A | N/A | N/A | ✓ | ✓ | ✓ | DR | N/A |
| (16) | ✓ | ✓ | ✓ | ✓ | ✓ | ✓ | ✓ | ✓ | ✓ | ✓ | ✓ | ✓ | ✓ | ✓ | ✓ | ✓ | ✓ | ✓ | ✓ | N/A |
| (17) | ✓ | ✓ | ✓ | ✓ | ✓ | ✓ | N/A | ✓ | ~ | ✓ | ✓ | ✓ | N/A | N/A | DR | ✓ | ✓ | ✓ | ✓ | ✓ |
| (18) | ✓ | ✓ | ✓ | ✓ | ✓ | X | X | ✓ | ✓ | ✓ | ✓ | ✓ | X | X | ✓ | ✓ | ✓ | ✓ | ✓ | ✓ |
| (19) | ✓ | ✓ | ✓ | ✓ | ✓ | ✓ | N/A | ✓ | ~ | ✓ | ✓ | ✓ | ✓ | X | DR | ✓ | ✓ | ✓ | ✓ | ✓ |

**Table S2.2:** CASP Critical Appraisal for included case-control studies.

|  | 1. | 2. | 3. | 4. | 5. | 6. | 7. | 8. | 9. | 10. | 11. | 12. |
| --- | --- | --- | --- | --- | --- | --- | --- | --- | --- | --- | --- | --- |
| (20) | ✓ | ✓ | ✓ | ✓ | ✓ | N/A | ✓ | N/A | N/A | ✓ | ✓ | ✓ |
| (21) | ✓ | ✓ | ✓ | ✓ | ✓ | N/A | ✓ | N/A | N/A | ✓ | ✓ | ✓ |
| (22) | ✓ | ✓ | ✓ | ✓ | ✓ | N/A | ✓ | N/A | N/A | ✓ | ✓ | ✓ |
| (23) | ✓ | ✓ | ✓ | ✓ | ✓ | N/A | ✓ | N/A | N/A | ✓ | ✓ | ✓ |

**Table S2.3*:*** CASP Critical Appraisal for included cohort studies.

|  | 1. | 2. | 3. | 4. | 5. | 6. | 7. | 8. | 9. | 10. | 11. | 12. | 13. | 14. |
| --- | --- | --- | --- | --- | --- | --- | --- | --- | --- | --- | --- | --- | --- | --- |
| (24) | ✓ | ✓ | ✓ | ✓ | ✓ | ✓ | ✓ | ✓ | N/A | ✓ | ✓ | ✓ | ✓ | N/A |
| (25) | ✓ | ✓ | ✓ | ✓ | ✓ | ✓ | ✓ | ✓ | N/A | ✓ | ✓ | ✓ | ✓ | N/A |
| (26) | ✓ | ✓ | ✓ | ✓ | ✓ | ✓ | ✓ | ✓ | N/A | ✓ | ✓ | ✓ | ✓ | N/A |
| (27) | ✓ | ✓ | ✓ | ✓ | ✓ | ✓ | ✓ | ✓ | N/A | ✓ | ✓ | ✓ | ✓ | N/A |
| (28) | ✓ | ✓ | ✓ | ✓ | ✓ | ✓ | ✓ | ✓ | N/A | ✓ | ✓ | ✓ | ✓ | N/A |
| (29) | ✓ | ✓ | ✓ | ✓ | X | X | ✓ | ✓ | N/A | DR | ✓ | ✓ | ✓ | N/A |
| (30) | ✓ | ✓ | ✓ | ✓ | ✓ | ✓ | ✓ | ✓ | N/A | ✓ | ✓ | ✓ | ✓ | N/A |
| (31) | ✓ | ✓ | ✓ | ✓ | ✓ | ✓ | ✓ | ✓ | N/A | ✓ | ✓ | ✓ | ✓ | N/A |
| (32) | ✓ | ✓ | ✓ | ✓ | X | X | ✓ | ✓ | N/A | ✓ | ✓ | ✓ | ✓ | N/A |
| (33) | ✓ | ✓ | ✓ | ✓ | ✓ | ✓ | ✓ | ✓ | N/A | ✓ | ✓ | ✓ | ✓ | N/A |
| (34) |  |  |  |  |  |  |  |  |  |  |  |  |  |  |
| (35) | ✓ | ✓ | ✓ | ✓ | ✓ | ✓ | ✓ | ✓ | N/A | ✓ | ✓ | ✓ | ✓ | N/A |
| (36) | ✓ | ✓ | ✓ | ✓ | X | X | ✓ | ✓ | N/A | ✓ | ✓ | ✓ | ✓ | N/A |
| (37) | ✓ | ✓ | ✓ | ✓ | X | X | ✓ | ✓ | N/A | ✓ | ✓ | ✓ | ✓ | N/A |
| (38) | ✓ | ✓ | ✓ | ✓ | ✓ | ✓ | ✓ | ✓ | N/A | ✓ | ✓ | ✓ | ✓ | N/A |
| (39) | ✓ | ✓ | ✓ | ✓ | ✓ | ✓ | ✓ | ✓ | N/A | ✓ | ✓ | ✓ | ✓ | N/A |
| (40) | ✓ | ✓ | ✓ | ✓ | X | X | ✓ | ✓ | N/A | DR | ✓ | ✓ | ✓ | N/A |
| (41) | ✓ | ✓ | ✓ | ✓ | ✓ | ✓ | ✓ | ✓ | N/A | ✓ | ✓ | ✓ | ✓ | N/A |
| (42) | ✓ | ✓ | ✓ | ✓ | ✓ | ✓ | ✓ | ✓ | N/A | ✓ | ✓ | ✓ | ✓ | N/A |
| (43) | ✓ | ✓ | ✓ | ✓ | X | X | ✓ | ✓ | N/A | DR | ✓ | ✓ | ✓ | N/A |
| (44) | ✓ | ✓ | ✓ | ✓ | ✓ | ✓ | ✓ | ✓ | N/A | ✓ | ✓ | ✓ | ✓ | N/A |
| (45) | ✓ | ✓ | ✓ | ✓ | X | X | ✓ | ✓ | N/A | ✓ | ✓ | ✓ | ✓ | N/A |
| (46) | ✓ | ✓ | ✓ | ✓ | X | X | ✓ | ✓ | N/A | ✓ | ✓ | ✓ | ✓ | N/A |
| (47) | ✓ | ✓ | ✓ | ✓ | ✓ | ✓ | N/A | ✓ | ✓ | ✓ | ✓ | ✓ | ✓ | N/A |
| (48) | ✓ | ✓ | ✓ | ✓ | ✓ | ✓ | N/A | ✓ | ✓ | ✓ | ✓ | ✓ | ✓ | N/A |
| (49) | ✓ | ✓ | ✓ | ✓ | X | X | ✓ | ✓ | N/A | ✓ | ✓ | ✓ | ✓ | N/A |
| (50) | ✓ | ✓ | ✓ | ✓ | ✓ | ✓ | X | ✓ | N/A | ✓ | ✓ | ✓ | ✓ | N/A |
| (51) | ✓ | ✓ | ✓ | ✓ | X | X | ✓ | ✓ | N/A | ✓ | ✓ | X | ✓ | N/A |
| (52) | ✓ | ✓ | ✓ | ✓ | X | X | ✓ | ✓ | N/A | DR | ✓ | ✓ | ✓ | N/A |

**Table S2.4*:*** CASP Quality Appraisal for included qualitative studies

|  | 1. | 2. | 3. | 4. | 5. | 6. | 7. | 8. | 9. | 10. |
| --- | --- | --- | --- | --- | --- | --- | --- | --- | --- | --- |
| (53) | ✓ | ✓ | ✓ | ✓ | ✓ | X | ✓ | ✓ | ✓ | ✓ |
| (54) | ✓ | ✓ | ✓ | ✓ | ✓ | X | ✓ | ✓ | ✓ | ✓ |

**Table S2.5:** CASP Quality Appraisal for included systematic reviews.

|  | 1. | 2. | 3. | 4. | 5. | 6. | 7. | 8. | 9. | 10. |
| --- | --- | --- | --- | --- | --- | --- | --- | --- | --- | --- |
| (55) | ✓ | ✓ | ✓ | ✓ | ✓ | N/A | ✓ | ✓ | ✓ | N/A |
| (56) | ✓ | ✓ | ✓ | ✓ | ✓ | N/A | ✓ | ✓ | ✓ | N/A |
| (57) | ✓ | ✓ | ✓ | X | N/A | N/A | N/A | ✓ | ✓ | N/A |

**S3**: PRISMA Checklist

| **Section/topic** | **#** | **Checklist item** | **Reported on page #** |
| --- | --- | --- | --- |
| **TITLE** | | |  |
| Title | 1 | Identify the report as a systematic review, meta-analysis, or both. | 1 |
| **ABSTRACT** | | |  |
| Structured summary | 2 | Provide a structured summary including, as applicable: background; objectives; data sources; study eligibility criteria, participants, and interventions; study appraisal and synthesis methods; results; limitations; conclusions and implications of key findings; systematic review registration number. | 2 |
| **INTRODUCTION** | | |  |
| Rationale | 3 | Describe the rationale for the review in the context of what is already known. | 3 |
| Objectives | 4 | Provide an explicit statement of questions being addressed with reference to participants, interventions, comparisons, outcomes, and study design (PICOS). | 3 |
| **METHODS** | | |  |
| Protocol and registration | 5 | Indicate if a review protocol exists, if and where it can be accessed (e.g., Web address), and, if available, provide registration information including registration number. | 4 |
| Eligibility criteria | 6 | Specify study characteristics (e.g., PICOS, length of follow-up) and report characteristics (e.g., years considered, language, publication status) used as criteria for eligibility, giving rationale. | 4 |
| Information sources | 7 | Describe all information sources (e.g., databases with dates of coverage, contact with study authors to identify additional studies) in the search and date last searched. | 4 |
| Search | 8 | Present full electronic search strategy for at least one database, including any limits used, such that it could be repeated. | S1 |
| Study selection | 9 | State the process for selecting studies (i.e., screening, eligibility, included in systematic review, and, if applicable, included in the meta-analysis). | 4 |
| Data collection process | 10 | Describe method of data extraction from reports (e.g., piloted forms, independently, in duplicate) and any processes for obtaining and confirming data from investigators. | 4, 5 |
| Data items | 11 | List and define all variables for which data were sought (e.g., PICOS, funding sources) and any assumptions and simplifications made. | 4, 5 |
| Risk of bias in individual studies | 12 | Describe methods used for assessing risk of bias of individual studies (including specification of whether this was done at the study or outcome level), and how this information is to be used in any data synthesis. | 5, S2 |
| Summary measures | 13 | State the principal summary measures (e.g., risk ratio, difference in means). | N/A |
| Synthesis of results | 14 | Describe the methods of handling data and combining results of studies, if done, including measures of consistency (e.g., I^2^) for each meta-analysis. | N/A |

Page 1 of 2

| **Section/topic** | **#** | **Checklist item** | **Reported on page #** |
| --- | --- | --- | --- |
| Risk of bias across studies | 15 | Specify any assessment of risk of bias that may affect the cumulative evidence (e.g., publication bias, selective reporting within studies). | S2 |
| Additional analyses | 16 | Describe methods of additional analyses (e.g., sensitivity or subgroup analyses, meta-regression), if done, indicating which were pre-specified. | N/A |
| **RESULTS** | | |  |
| Study selection | 17 | Give numbers of studies screened, assessed for eligibility, and included in the review, with reasons for exclusions at each stage, ideally with a flow diagram. | 5 |
| Study characteristics | 18 | For each study, present characteristics for which data were extracted (e.g., study size, PICOS, follow-up period) and provide the citations. | 5, 6 |
| Risk of bias within studies | 19 | Present data on risk of bias of each study and, if available, any outcome level assessment (see item 12). | S2 |
| Results of individual studies | 20 | For all outcomes considered (benefits or harms), present, for each study: (a) simple summary data for each intervention group (b) effect estimates and confidence intervals, ideally with a forest plot. | N/A |
| Synthesis of results | 21 | Present results of each meta-analysis done, including confidence intervals and measures of consistency. | N/A |
| Risk of bias across studies | 22 | Present results of any assessment of risk of bias across studies (see Item 15). | S2 |
| Additional analysis | 23 | Give results of additional analyses, if done (e.g., sensitivity or subgroup analyses, meta-regression [see Item 16]). | N/A |
| **DISCUSSION** | | |  |
| Summary of evidence | 24 | Summarize the main findings including the strength of evidence for each main outcome; consider their relevance to key groups (e.g., healthcare providers, users, and policy makers). | 9 |
| Limitations | 25 | Discuss limitations at study and outcome level (e.g., risk of bias), and at review-level (e.g., incomplete retrieval of identified research, reporting bias). | 10 |
| Conclusions | 26 | Provide a general interpretation of the results in the context of other evidence, and implications for future research. | 9, 10, 11 |
| **FUNDING** | | |  |
| Funding | 27 | Describe sources of funding for the systematic review and other support (e.g., supply of data); role of funders for the systematic review. | 1 |

References

1. Beard E, Brown J, West R, Angus C, Brennan A, Holmes J, et al. Deconstructing the Alcohol Harm Paradox: A population based survey of adults in England. PLoS One. 2016;11(9):1–17.

2. Bellis MA, Hughes K, Nicholls J, Sheron N, Gilmore I, Jones L. The alcohol harm paradox: Using a national survey to explore how alcohol may disproportionately impact health in deprived individuals. BMC Public Health [Internet]. 2016;16(1):1–10. Available from: http://dx.doi.org/10.1186/s12889-016-2766-x

3. Breakwell C, Baker A, Griffiths C, Jackson G, Fegan G, Marshall D. Trends and geographical variations in alcohol-related deaths in the United Kingdom, 1991-2004. Health Stat Q. 2007;(33):6–24.

4. Brown RL, Richman JA, Rospenda KM. Economic stressors and alcohol-related outcomes: Exploring gender differences in the mediating role of somatic complaints. J Addict Dis. 2014;33(4):303–13.

5. Connor JL, Kypri K, Bell ML, Cousins K. Alcohol outlet density, levels of drinking and alcohol-related harm in New Zealand: A national study. J Epidemiol Community Health. 2010;65(10):841–6.

6. Karriker-Jaffe KJ, Roberts SCM, Bond J. Income inequality, alcohol use, and alcohol-related problems. Am J Public Health. 2013;103(4):649–56.

7. Lewer D, Meier P, Beard E, Boniface S, Kaner E. Unravelling the alcohol harm paradox: A population-based study of social gradients across very heavy drinking thresholds. BMC Public Health [Internet]. 2016;16(1):1–11. Available from: http://dx.doi.org/10.1186/s12889-016-3265-9

8. Livingston M. Socioeconomic differences in alcohol-related risk-taking behaviours. Drug Alcohol Rev. 2014;33(6):588–95.

9. Jonas H, Dietze P, Rumbold G, Hanlin K, Cvetkovski S, Laslett AM. Associations between alcohol related hospital admissions and alcohol consumption in Victoria: Influence of socio-demographic factors. Aust N Z J Public Health. 1999;23(3):272–9.

10. Karriker-Jaffe KJ, Zemore SE, Mulia N, Jones-Webb R, Bond J, Greenfield TK. Neighborhood disadvantage and adult alcohol outcomes: Differential risk by race and gender. J Stud Alcohol Drugs. 2012;73(6):865–73.

11. Romelsjö A, Lundberg M. The changes in the social class distribution of moderate and high alcohol consumption and of alcohol-related disabilities over time in Stockholm County and in Sweden. Addiction. 1996;91(9):1307–24.

12. Sadler S, Angus C, Gavens L, Gillespie D, Holmes J, Hamilton J, et al. Understanding the alcohol harm paradox: an analysis of sex- and condition-specific hospital admissions by socio-economic group for alcohol-associated conditions in England. Addiction. 2017;112(5):808–17.

13. Van Oers JAM, Bongers IMB, Van De Goor LAM, Garretsen HFL. Alcohol consumption, alcohol-related problems, problem drinking, and socioeconomic status. Alcohol Alcohol. 1999;34(1):78–88.

14. Huckle T, You RQ, Casswell S. Socio-economic status predicts drinking patterns but not alcohol-related consequences independently. Addiction. 2010;105(7):1192–202.

15. Kuendig H, Plant ML, Plant MA, Kuntsche S, Miller P, Gmel G, et al. Beyond drinking: Differential effects of demographic and socioeconomic factors on alcohol-related adverse consequences across European countries. Eur Addict Res. 2008;14(3):150–60.

16. Mulia N, Zemore SE. Social adversity, stress, and alcohol problems: Are racial/ethnic minorities and the poor more vulnerable? J Stud Alcohol Drugs. 2012;73(4):570–80.

17. Thor S, Karlsson P, Landberg J. Social Inequalities in Harmful Drinking and Alcohol-Related Problems among Swedish Adolescents. Alcohol Alcohol. 2019;54(5):532–9.

18. Skogen JC, Bøe T, Thørrisen MM, Riper H, Aas RW. Sociodemographic characteristics associated with alcohol consumption and alcohol-related consequences, a latent class analysis of the Norwegian WIRUS screening study. BMC Public Health. 2019;19(1):1–12.

19. Møller SP, Pisinger VSC, Christensen AI, Tolstrup JS. Socioeconomic position and alcohol-related harm in Danish adolescents. J Epidemiol Community Health. 2019;73(9):839–45.

20. Boyle T, Fritschi L, Tabatabaei SM, Ringwald K, Heyworth JS. Smoking, alcohol, diabetes, obesity, socioeconomic status and the risk of colorectal cancer in a population-based case-control study. Cancer Causes Control. 2014;25(12):1659–68.

21. Conway DI, Brenner DR, McMahon AD, Macpherson LMD, Agudo A, Ahrens W, et al. Estimating and explaining the effect of education and income on head and neck cancer risk: INHANCE consortium pooled analysis of 31 case-control studies from 27 countries. Int J Cancer. 2015;136(5):1125–39.

22. Menvielle G, Luce D, Goldberg P, Leclerc A. Smoking, alcohol drinking, occupational exposures and social inequalities in hypopharyngeal and laryngeal cancer. Int J Epidemiol. 2004;33(4):799–806.

23. Stanford-Moore G, Bradshaw PT, Weissler MC, Zevallos JP, Brennan P, Anantharaman D, et al. Interaction between known risk factors for head and neck cancer and socioeconomic status: the Carolina Head and Neck Cancer Study. Cancer Causes Control [Internet]. 2018;29(9):863–73. Available from: http://dx.doi.org/10.1007/s10552-018-1062-8

24. Backhans MC, Balliu N, Lundin A, Hemmingsson T. Unemployment is a risk factor for hospitalization due to alcohol problems: A longitudinal study based on the Stockholm Public Health Cohort (SPHC). J Stud Alcohol Drugs. 2016;77(6):936–42.

25. Degerud E, Ariansen I, Ystrom E, Graff-Iversen S, Høiseth G, Mørland J, et al. Life course socioeconomic position, alcohol drinking patterns in midlife, and cardiovascular mortality: Analysis of Norwegian population-based health surveys. PLoS Med. 2018;15(1):1–20.

26. Evans-Polce RJ, Staff J, Maggs JL. Alcohol abstention in early adulthood and premature mortality: Do early life factors, social support, and health explain this association? Soc Sci Med [Internet]. 2016;163:71–9. Available from: http://dx.doi.org/10.1016/j.socscimed.2016.06.052

27. Gartner A, Trefan L, Moore S, Akbari A, Paranjothy S, Farewell D. Drinking beer, wine or spirits - Does it matter for inequalities in alcohol-related hospital admission? A record-linked longitudinal study in Wales. BMC Public Health. 2019;19(1):1–13.

28. Glei DA, Lee C, Weinstein M. Socioeconomic disparities in U.S. mortality: The role of smoking and alcohol/drug abuse. SSM - Popul Heal [Internet]. 2020;12:100699. Available from: https://doi.org/10.1016/j.ssmph.2020.100699

29. Herttua K, Mäkelä P, Martikainen P. Differential trends in alcohol-related mortality: A register-based follow-up study in Finland in 1987-2003. Alcohol Alcohol. 2007;42(5):456–64.

30. Lawder R, Grant I, Storey C, Walsh D, Whyte B, Hanlon P. Epidemiology of hospitalization due to alcohol-related harm: Evidence from a Scottish cohort study. Public Health [Internet]. 2011;125(8):533–9. Available from: http://dx.doi.org/10.1016/j.puhe.2011.05.007

31. Lundin A, Backhans M, Hemmingsson T. Unemployment and Hospitalization Owing to an Alcohol-Related Diagnosis Among Middle-Aged Men in Sweden. Alcohol Clin Exp Res. 2012;36(4):663–9.

32. Mäkelä P, Paljärvi T. Do consequences of a given pattern of drinking vary by socioeconomic status? a mortality and hospitalisation follow-up for alcohol-related causes of the Finnish Drinking Habits Surveys. J Epidemiol Community Health. 2008;62(8):728–33.

33. Mulia N, Karriker-Jaffe KJ. Interactive influences of neighborhood and individual socioeconomic status on alcohol consumption and problems. Alcohol Alcohol. 2012;47(2):178–86.

34. Nielsen NR, Schnohr P, Jensen G, Grønbæk M. Is the relationship between type of alcohol and mortality influenced by socio-economic status? J Intern Med. 2004;255(2):280–8.

35. Salom CL, Williams GM, Najman JM, Alati R. Does early socio-economic disadvantage predict comorbid alcohol and mental health disorders? Drug Alcohol Depend [Internet]. 2014;142:146–53. Available from: http://dx.doi.org/10.1016/j.drugalcdep.2014.06.011

36. Shaper AG, Wannamethee G, Walker M. Alcohol and Mortality in British Men: Explaining the U-Shaped Curve. Lancet. 1988;332(8623):1267–73.

37. Singh GK, Hoyert DL. Social Epidemiology of Chronic Liver Disease and Cirrhosis Mortality in the United States, 1935–1997: Trends and Differentials by Ethnicity, Socioeconomic Status, and Alcohol Consumption [Internet]. Vol. 72, Human Biology. Wayne State University Press; 2000 [cited 2020 Apr 8]. p. 801–20. Available from: https://www.jstor.org/stable/41465881

38. Stewart D, Han L, Doran T, McCambridge J. Alcohol consumption and all-cause mortality an analysis of general practice database records for patients with long-term conditions. Journal of epidemiology and community health.; 2017.

39. Sydén L, Sidorchuk A, Mäkelä P, Landberg J. The contribution of alcohol use and other behavioural, material and social factors to socio-economic differences in alcohol-related disorders in a Swedish cohort. Addiction. 2017;112(11):1920–30.

40. Whitley E, Batty GD, Hunt K, Popham F, Benzeval M. The role of health behaviours across the life course in the socioeconomic patterning of all-cause mortality: The west of Scotland twenty-07 prospective cohort study. Ann Behav Med. 2014;47(2):148–57.

41. Katikireddi SV, Whitley E, Lewsey J, Gray L, Leyland AH. Socioeconomic status as an effect modifier of alcohol consumption and harm: analysis of linked cohort data. Lancet Public Heal [Internet]. 2017;2(6):e267–76. Available from: http://dx.doi.org/10.1016/S2468-2667(17)30078-6

42. McDonald SA, Hutchinson SJ, Bird SM, Graham L, Robertson C, Mills PR, et al. Association of self-reported alcohol use and hospitalization for an alcohol-related cause in Scotland: A record-linkage study of 23 183 individuals. Addiction. 2009;104(4):593–602.

43. Norström T, Romelsjö A. Social class, drinking and alcohol-related mortality. J Subst Abuse. 1998;10(4):385–95.

44. Nweze IC, DiGiacomo JC, Shin SS, Gupta C, Ramakrishnan R, Angus LDG. Demographic and socioeconomic factors influencing disparities in prevalence of alcohol-related injury among underserved trauma patients in a safety-net hospital. Injury [Internet]. 2016;47(12):2635–41. Available from: http://dx.doi.org/10.1016/j.injury.2016.10.020

45. Roberts SE, Williams JG, Meddings D, Goldacre MJ. Incidence and case fatality for acute pancreatitis in England_ Geographical variation, social deprivation, alcohol consumption and aetiology - a record linkage study. Alimentary Pharmacology and Therapeutics. 2008. p. 931–41.

46. Roberts SE, Akbari A, Thorne K, Atkinson M, Evans PA. The incidence of acute pancreatitis: Impact of social deprivation, alcohol consumption, seasonal and demographic factors. Aliment Pharmacol Ther. 2013;38(5):539–48.

47. Peña S, Mäkelä P, Härkänen T, Heliövaara M, Gunnar T, Männistö S, et al. Alcohol-related Harm Measurement error as an explanation for the alcohol harm paradox: analysis of eight cohort studies. Int J Epidemiol [Internet]. 2020 [cited 2021 Feb 11];1836–46. Available from: https://academic.oup.com/ije/article/49/6/1836/5913111

48. Peña S, Mäkelä P, Laatikainen T, Härkänen T, Männistö S, Heliövaara M, et al. Joint effects of alcohol use, smoking and body mass index as an explanation for the alcohol harm paradox: causal mediation analysis of eight cohort studies. Addiction. 2021;

49. Trias-Llimós S, Bosque-Prous M, Obradors-Rial N, Teixidó-Compañó E, Belza MJ, Janssen F, et al. Alcohol and educational inequalities: Hazardous drinking prevalence and all-cause mortality by hazardous drinking group in people aged 50 and older in Europe. Subst Abus [Internet]. 2020;0(0):1–9. Available from: https://doi.org/10.1080/08897077.2020.1773597

50. Thern E, Ramstedt M, Svensson J. The associations between unemployment at a young age and binge drinking and alcohol-related problems. Eur J Public Health. 2019;30(2):368–73.

51. Rhew IC, Duckworth JC, Hurvitz PM, Lee CM. Within- and between-person associations of neighborhood poverty with alcohol use and consequences: A monthly study of young adults. Drug Alcohol Depend [Internet]. 2020;212(January):108068. Available from: https://doi.org/10.1016/j.drugalcdep.2020.108068

52. Norström T, Landberg J. The link between per capita alcohol consumption and alcohol-related harm in educational groups. Drug Alcohol Rev. 2020;39(6):656–63.

53. Parkman T, Neale J, Day E, Drummond C. Qualitative exploration of why people repeatedly attend emergency departments for alcohol-related reasons. BMC Health Serv Res. 2017;17(1):1–9.

54. Hart A. Assembling Interrelations Between Low Socioeconomic Status and Acute Alcohol-Related Harms Among Young Adult Drinkers. Contemp Drug Probl. 2015;42(2):148–67.

55. Jones L, Bates G, McCoy E, Bellis MA. Relationship between alcohol-attributable disease and socioeconomic status, and the role of alcohol consumption in this relationship: A systematic review and meta-analysis. BMC Public Health. 2015;15(1).

56. Probst C, Kilian C, Sanchez S, Lange S, Rehm J. The role of alcohol use and drinking patterns in socioeconomic inequalities in mortality: a systematic review. Lancet Public Heal [Internet]. 2020;5(6):e324–32. Available from: http://dx.doi.org/10.1016/S2468-2667(20)30052-9

57. Roche A, Kostadinov V, Fischer J, Nicholas R, O’Rourke K, Pidd K, et al. Addressing inequities in alcohol consumption and related harms. Health Promot Int. 2015;30:ii20–35.
